# Clinical trial protocol of the ASTER trial: a double-blind, randomized, placebo-controlled phase III trial evaluating the use of acetylsalicylic acid (ASA) for enhanced early detection of colorectal neoplasms

**DOI:** 10.1186/s12885-018-4826-3

**Published:** 2018-09-24

**Authors:** Kaja Tikk, David Czock, Walter E. Haefeli, Annette Kopp-Schneider, Hermann Brenner

**Affiliations:** 1 Division of Clinical Epidemiology and Aging Research, German Cancer Research Center (DKFZ), Im Neuenheimer Feld 581, 69120 Heidelberg, Germany; 2 German Cancer Consortium (DKTK), German Cancer Research Center (DKFZ), Heidelberg, Germany; 3 Department of Clinical Pharmacology and Pharmacoepidemiology, University Hospital Heidelberg, Heidelberg, Germany; 4 Department of Biostatistics, German Cancer Research Center (DKFZ), Heidelberg, Germany; 5 Division of Preventive Oncology, German Cancer Research Center (DKFZ) and National Center for Tumor Diseases (NCT), Heidelberg, Germany

**Keywords:** Colorectal cancer, Early detection, Screening, Acetylsalicylic acid, Sensitivity, Specificity

## Abstract

**ᅟ:**

Immunochemical fecal occult blood tests (iFOBTs) are increasingly used for colorectal cancer (CRC) screening. In our preceding observational study, sensitivity for detecting advanced colorectal neoplasms by iFOBT was 70.8% among users of low-dose acetylsalicylic acid compared with 35.9% among non-users (*p* = 0.001), whereas there were only very small differences in specificity. In receiver operating characteristics (ROC) analyses, the area under the curve (AUC) was much higher for acetylsalicylic acid users than for non-users, with particularly strong differences in men (0.87 versus 0.68, *p* = 0.003). These findings suggested that use of acetylsalicylic acid before conduct of iFOBT might be a promising approach to improve non-invasive screening for CRC.

**Methods/design:**

In this randomized, double-blind, placebo-controlled trial, the diagnostic performance of two iFOBTs for detecting advanced colorectal neoplasms after a single low-dose of acetylsalicylic acid (300 mg) compared to placebo is evaluated. Acetylsalicylic acid or placebo is administered at least 5 days before a planned, study-independent colonoscopic screening in 2400 participants aged 40 to 80 years. Stool samples are obtained before and on three different days after the single dose of acetylsalicylic acid or placebo. In addition, optional blood samples are taken for future biomarker analyses. The diagnostic performance of the iFOBTs will be compared to the results of the colonoscopy as a gold standard for the diagnosis of colorectal neoplasms. Additionally, gender-specific performance of the tests and gain in diagnostic performance by test application on multiple days will be evaluated.

**Discussion:**

If the findings from our preceding observational study will be confirmed in this large trial, the proposed low-risk, inexpensive intervention would considerably improve the diagnostic accuracy of iFOBTs and thus lead to enhanced early detection of colorectal neoplasms. Thus, the results of this trial may have a large public health impact.

**Trial registration:**

This trial was registered before recruitment of the participants in www.clinicaltrialsregister.eu on the 30th of May 2012: EudraCT No.: 2011–005603-32 and in www.drks.de on 13th of March 2012: German Clinical Trials Register DRKS-ID: DRKS00003252.

**Electronic supplementary material:**

The online version of this article (10.1186/s12885-018-4826-3) contains supplementary material, which is available to authorized users.

## Background

Colorectal cancer (CRC) is the third most common cancer and the fourth most common cancer cause of death globally, with more than 1.3 million new cases and more than 700,000 deaths annually [1]. In Germany, there are over 60,000 new cases and around 25,000 deaths due to CRC per year [2]. The commonly slow progression from detectable and fully curable precursors (adenomas) to still-curable early-stage CRC to advanced CRC provides promising perspectives for prevention and early detection by screening.

Randomized studies have demonstrated that CRC incidence and CRC-associated mortality can be reduced by 20–30% through annual or biennial screening with fecal occult blood tests (FOBTs) [3–5], despite the low sensitivity for detection of colorectal adenomas of conventional (guaiac based) FOBTs. Substantially higher sensitivity for detection of colorectal adenomas can be achieved with newer, immunochemical FOBTs (iFOBTs), which are increasingly recommended and employed for non-invasive CRC screening. Although iFOBTs detect approximately four out of five CRCs [6], the sensitivity for detecting advanced adenomas, the precursors of most CRCs, still remains far below 50% at cut points yielding 90–95% specificity required for population-based screening. Thus, there is an unmet medical need for easy-to-implement screening methods for detection of high-risk colorectal adenomas in addition to CRC.

In our preceding prospective screening study conducted among 1979 participants of screening colonoscopy in Germany, the sensitivity for detecting advanced colorectal neoplasms (i.e. either CRC or advanced adenoma) by iFOBT was 70.8% among users of low-dose acetylsalicylic acid compared with 35.9% among non-users (*p* = 0.001), whereas there were only very small differences in specificity (85.7% for users compared with 89.2 to 91.1% for non-users) [7]. In receiver operating characteristics (ROC) analyses, the area under the curve (AUC) was much higher among users than among non-users, with differences being particularly pronounced among men (0.87 versus 0.68, *p* = 0.003). This suggests that use of acetylsalicylic acid prior to conduct of iFOBT might be a particularly promising approach for enhancing non-invasive screening for CRC. It is hypothesized that the underlying mechanism could be an enhanced tendency of (micro-)bleeding of colorectal neoplasms.

### Objectives

The main objective of this trial is to evaluate the diagnostic performance (sensitivity, specificity, positive and negative predictive values, likelihood ratios, and AUC of two iFOBTs for detecting advanced colorectal neoplasms after a single dose of acetylsalicylic acid as compared to placebo.

Secondary objectives are: 1) To study gender-specific performance of the two iFOBTs and the possible gain in diagnostic performance by stool sampling on multiple days; 2) To study the safety of single-dose acetylsalicylic acid in the selected population; 3) To collect blood samples for additional biomarker analyses (optional).

## Methods/Design

### Study design

This trial is designed as a randomized, double-blind, placebo-controlled, phase III multicenter trial (German title: ‘Mit **AS**S Darm**t**umore früher **er**kennen’, acronym ASTER).

### Screening

**Fig. 1:**
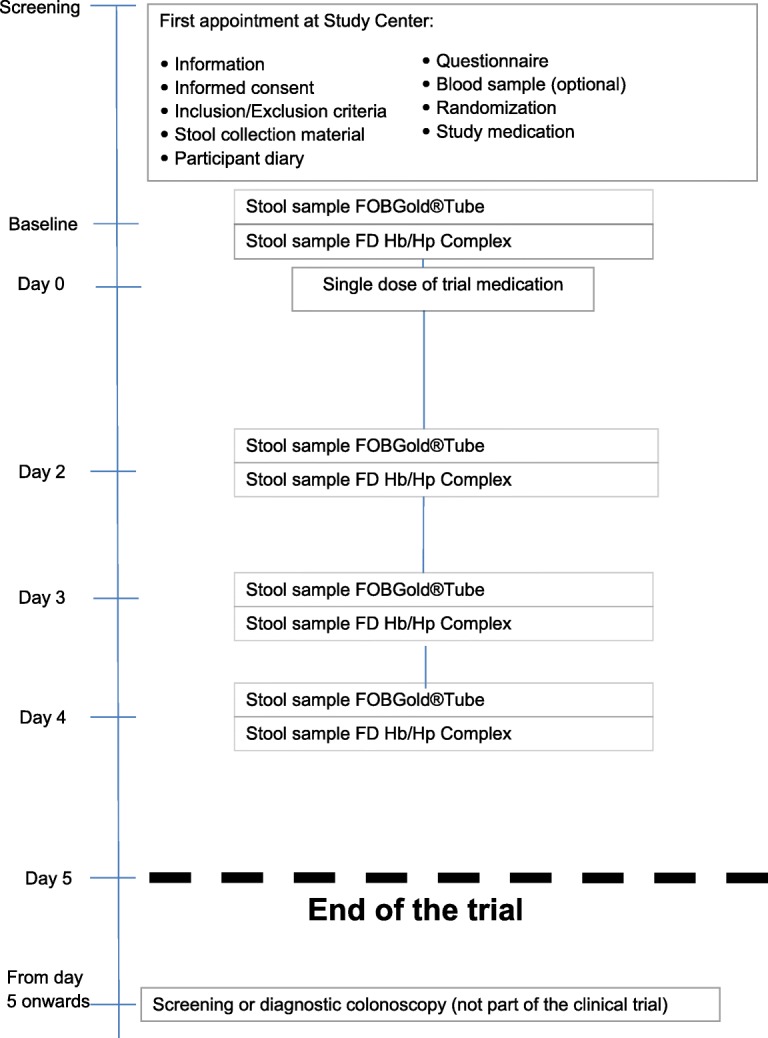
Overview of study flow

In total, 2400 eligible participants aged 40 to 80 years and with no recent use of acetylsalicylic acid are recruited when visiting one of the collaborating gastroenterology private practices or hospitals (hereinafter referred to as ‘study centers’) for an informative appointment, which is routinely scheduled a few weeks before colonoscopy. The study flow of the trial is illustrated in Fig. 1. The exact schedule can be found in Table 1. In addition to participants scheduled for a screening colonoscopy, patients visiting the study centers for a diagnostic colonoscopy meeting the inclusion criteria are also asked to participate. Thus, the colonoscopy procedure is planned beforehand and conducted independently from our trial.

**Table 1:** Study schedule

Trial-specific procedures	Screening	Baseline	Treat-ment	Observation			Post-study
Timeline			Day 0	Day 2	Day 3	Day 4	Colonoscopy from day 5 onwards
Informed consent	X						
Inclusion/ exclusion criteria	X						
Randomization	X						
Physical examination	X						
Patient parameters^a^	X						
Blood sampling for biomarker analyses (optional)^e^	X						
Questionnaire^b^	X						
Handing over stool collection material and subject diary^c^	X						
Drug administration (Acetylsalicylic acid or placebo)			X				
Stool sample^d^ (FOBGold®Tube Screen)		X		X	X	X	
Stool sample^d^ (FD Hb/Hp Complex)		X		X	X	X	
Concomitant therapy recording			X	X	X	X	
(S)AE recording^f^			X	X	X	X	

^a^Age, sex, weight, height, and blood pressure

^b^The questionnaire includes questions on family history of CRC and lifestyle factors such as smoking and alcohol consumption

^c^Eight kits are handed over

^d^If stool collection on these days is not possible because of constipation or other reasons, stool collection may be postponed to the subsequent days; participants are also allowed to collect only two stool samples, which should be at baseline (without medication) and on day 2, if possible

^e^Blood sampling for biomarker analysis: 2 EDTA tubes; 2 serum tubes

^f^Interview of participants before colonoscopy

During the first appointment (day of Screening), participants are informed about the trial, receive the printed study information, and have the opportunity to ask questions. A detailed medical and drug history is taken and inclusion and exclusion criteria are checked (see the full list of inclusion and exclusion criteria for details in Appendix). Women who are not postmenopausal must have a negative pregnancy test before signing consent. If all inclusion criteria are met, no exclusion criteria are present, and the patient consents to the blood sampling, four extra tubes of blood (2 EDTA tubes and 2 serum tubes) are taken for analysis of additional biomarkers (optional). In addition, patients receive four iFOBT kits for each of two different iFOBTs (FOBGold®Tube Screen, an internationally widely used quantitative iFOBT [8, 9], as well as the FD Hb/Hp Complex quick test, a qualitative chromatographic test from Frost Diagnostika; hence, eight stool kits in total are dispensed along with instructions on when and how to use them and including a device that is hung in the toilet and aids in easy collection of the stool samples.

### Study medication

Participants are randomized to receive either a single dose of 300 mg acetylsalicylic acid (without enteric coating, Ratiopharm GmbH) or placebo (containing cellulose, lactose and magnesium stearate; Winthrop Arzneimittel GmbH); thereby, a unique participant number is generated and documented on the day of screening in the participants’ log.

Both, participants and their physicians are blinded with respect to the study medication.

### Stool collection

Participants are asked to collect a baseline stool sample before taking the study medication. If the participant cannot collect a stool sample at baseline (e.g. due to constipation), this stool sample is skipped and the study medication is taken without a baseline stool sample. Further stool samples are collected from day 2 onwards on 3 different, preferably consecutive days (day 2, 3, and 4). If stool collection on these days is not possible because of constipation or other reasons, stool collection may be postponed to the subsequent day. Participants are still eligible for inclusion in the analysis if they only provide two stool samples (before taking study medications and on day 2, if possible). At each time point at which stool samples are collected, one kit of each test is used and the actual day documented. The stool sampling devices ensure collection of defined volumes of stool which are given to a buffer that hinders degradation of hemoglobin (for example FOBGold®Tube Screen test: 10 mg stool in 1.7 ml buffer). All stool samples are collected at the participants’ homes and prior to initiation of large bowel preparation for colonoscopy. The stool samples are stored in the refrigerators and sent by mail to the coordinating center (Heidelberg) using special pre-paid and addressed mailing devices.

### Participants’ information

To ensure adherence to the study medication, participants are instructed in detail before start of the trial. Given that the medication scheme includes only one single dose of study drug, no major problems in taking the medication is expected. All participants receive a participant diary in which they document if and when they took the study medication, and if and when they collected the stool samples. The participant sends this diary to the coordinating center in Heidelberg using a prepaid envelop. Finally, participants are asked to fill out a standardized questionnaire addressing potential determinants of risk of colorectal neoplasms and of test performance, including general participant characteristics, co-morbidities, and lifestyle factors. They can either do this during their visit to the study center or at home.

### Colonoscopy

The colonoscopy is planned and conducted in the setting of screening for CRC or diagnostic workup and, as such, it is not part of the trial. However, the investigator must ensure that there are at least 5 days (4 full acetylsalicylic acid-free days) between taking the study medication and the colonoscopy. Although a single dose of acetylsalicylic acid is not considered a contraindication for elective colonoscopy, this time interval was chosen to minimize any possibility of an increased risk of bleeding at colonoscopy or endoscopic removal of small polyps [10–12]. However, the findings from screening colonoscopy are collected and used in the analysis.

Colonoscopy (and histology) reports are de-identified (i.e. any personal data is removed and the participant number is noted) and sent from the study centers to the coordinating center (Division of Clinical Epidemiology and Aging Research at the German Cancer Research Centre, Heidelberg).

### Data collection and documentation in recruiting centers

For each participant in the clinical trial written informed consent is obtained before enrollment into the trial, which is stored by the investigator in the investigator site file (ISF) at study centers for at least 10 years after the end of the trial.

All participants’ data collected at the study centers are entered into the electronic Case Report Form (eCRF) by the study physicians and physician assistants on site. Data collected in the eCRF includes: participant number (available once a patient is randomized); participant’s medical history, and data collected during physical examination; verification of compliance with inclusion and exclusion criteria; dates of participation in the trial, including date of informed consent, dispensing of study material, and date of colonoscopy; information on whether an optional blood sample was taken; laboratory values (thrombocytes) if available from clinical routine; adverse events (AEs); protocol violations; date of drop-out and reasons, if applicable.

The eCRF does not contain the participant’s identifying information, but only his/her participant number. Entries in the eCRF may only be made by the investigator or persons authorized by him. If corrections in the eCRF are necessary, the initial entry will be kept in an electronic log (audit-trail). A list is kept of the individuals who are authorized to make data changes. At the end of the trial, data collected in the eCRF are transferred digitally into a database at the coordinating center in Heidelberg.

To confirm adherence to the study medication, the participants are asked to bring the empty package of the study medication when coming to the planned colonoscopy. Information on medication usage is collected in a drug accountability log.

### Data collection and documentation in the coordinating center

The linkage between biological samples, questionnaires, and colonoscopy results is established only by the unique participant number. The information collected from colonoscopy and histology reports is entered into an additional standardized study database by trained staff in the coordinating center, using double data entry by two independent and blinded staff members. Data entries are checked for inconsistencies through comparison of the corresponding data sets. In case of differences in data sets, original reports are checked for validation.

Documentation of information collected in the questionnaire and participants’ diary include automated scanning of the questionnaires, optical verification of the scans by trained staff, and comprehensive plausibility checks prior to statistical analysis. All collected information is stored at the coordinating center at least 10 years after the end of the trial.

### Analysis of stool samples

Laboratory analyses of immunochemical FOBGold®Tube Screen test are performed in a blinded manner in an external cooperating laboratory (Limbach Laboratory, Heidelberg). Analyses for the FD Hb/Hp Complex test are performed at the laboratory of the Department of Clinical Epidemiology and Aging Research at the German Cancer research Center in Heidelberg. The FD Hb/Hp Complex quick test enables to test human hemoglobin (Hb) and hemoglobin/haptoglobin complex (Hb/Hp); however, in this trial, only the results from the Hb test will be reported. Both laboratories have extensive experience in the analysis of iFOBTs from previous studies of this group. The iFOBTs used in this trial are commercially available, validated tests. Standard Operating Procedures are established and followed for the analyses.

### Analysis and storage of blood samples

Blood samples are picked up from the study centers by a lab sample transportation service and brought to the coordinating center in Heidelberg. Blood samples are stored at the coordinating center at − 80 °C without a time limit for future development and analysis of biomarkers including genetic markers potentially related to the presence of advanced adenomas and/or colorectal carcinoma and for analysis of the determinants of the effects of acetylsalicylic acid.

As intensive research into new blood tests is continuously ongoing, it is neither meaningful nor possible to explicitly state which exact markers will be tested in the future. Long-term storage of the blood samples collected in this trial will enable timely validation of emerging promising early detection markers in the years to come.

Only the staff of the coordinating center will have access to the samples. However, the samples may be transported to laboratories of cooperating partners (including international partners) for specific analyses.

All laboratory analyses are done in a blinded fashion with respect to both treatment given and clinical/colonoscopy data.

### Quality assurance

The study medication (both acetylsalicylic acid and placebo) is packaged, labeled and blinded according to applicable GCP-V and GMP rules by the Pharmacy of the University Hospital Heidelberg, holding a manufacturing authorization.

There is a responsible investigator in every recruiting study center. The responsible investigator has completed a certified investigator training, including training in ICH-GCP, GCP-V, and AMG (German drug law). The responsible investigator of a study center keeps a confidential list with full names and dates of birth of all participants in the trial, giving reference to the participant’s records. This list is kept in the investigator site file at the study centers. For enrolled participants the date of enrolment and the allocation/randomization number is recorded in this list. The identity of the participants will not be revealed to unauthorized persons.

The participating study centers are visited on a regular basis by qualified staff (visits include an initiation visit, a number of interim visits during the recruitment phase (on-site-monitoring), and a close-out visit). A report is written after each visit.

The steering committee (consisting of the head of the coordinating center, the clinical pharmacology consultant, and the principal investigator of the study (LKP)) meets regularly, either in person or via a telephone conference, to discuss study progress, monitoring reports, and any study-related problems. The conclusions of these meetings are documented.

### Safety assessment

Participants are asked about concurrent use of medication and diseases at inclusion to minimize the risk of AEs. Candidate participants in the trial are excluded if they use potentially interacting medications or have illnesses that may be worsened by participation in this trial. In the patient information documents, participants are informed about all relevant potential AEs that may occur. Furthermore, participants are instructed to contact their general practitioner or the investigator in the unlikely case of a severe medical problem.

The observation period for AEs is defined as day 0 to day 4 for this trial. When the participants come for the scheduled study-independent colonoscopy, they are asked about any potential AEs or events potentially related to the use of the medication. All AEs are documented and the investigator judges intensity, seriousness, and relatedness of an AE using standard criteria. This information is documented in a standardized way in the eCRF. All serious AEs (SAEs) must be reported within 24 h, or on the next working day at the latest, using a standardized SAE report form.

### Methods against bias

The trial is randomized to account for potential confounding factors. Block randomization is used by randomizing participants within blocks such that an equal number is assigned to each treatment (the order in which treatments are allocated in each block is random). Randomization list is compiled with the randomization software RITA – Randomisation in Treatment Arms, Version 1.24, it.e.c.x.

All laboratory tests as well as extraction of clinical data from colonoscopy and pathology reports are done in a blinded manner to avoid information bias. Data extraction and data entry (where applicable) is done by two independent and blinded reviewers. Discrepant coding is resolved according to standard operating procedures to achieve the maximum accuracy possible. In addition, the study centers as well as all participants receive detailed instructions to ensure uniform collection and handling of stool and blood samples. Pre-analysis conditions, including data on storage conditions and duration and mode of transportation, are also documented in detail to control for potential variation.

### Sample size calculation

The sample size is estimated based on the results of the preliminary study including participants from CRC screening with self-reported acetylsalicylic acid use [7]. Accounting for an expected loss of data of about 20% and with an expected prevalence of advanced neoplasms of 10%, 100 advanced neoplasms are expected in each of the two groups. Assuming a sensitivity of 36% in the placebo group (as in our preliminary study) and applying 2-sided chi-square tests with continuity correction at an alpha level of 0.05, this trial should have a power of 90% to detect an increase in sensitivity by short term use of low-dose acetylsalicylic acid to 60% in the entire trial population, and a power of 90% to detect an increase in sensitivity to 70% in gender-specific analyses (sensitivity was 71% in our preliminary study [7]). Furthermore, specificity will be estimated at high levels of precision in both groups, with an expected confidence interval ranging from 88 to 92% for a specificity of 90%.

### Statistical analysis

Standard techniques for the analysis of diagnostic tests will be used [13, 14]. Diagnostic accuracy of the two iFOBTs at the different time points of stool collection will be quantified by estimating sensitivity, specificity, likelihood ratios, positive and negative predictive values (and corresponding 95% confidence intervals) at cut points yielding levels of specificity typically required for routine testing of average-risk populations (> 90%), using the results of colonoscopy as the gold standard. These results will be compared between participants randomized to receive acetylsalicylic acid and those randomized to receive placebo in an intention-to-treat analysis. For the primary endpoint, sensitivity for predefined (test-specific) cut points of test positivity of the FOBGold®Tube Screen test will be compared between users and non-users of acetylsalicylic acid. The stool samples taken on day 2 will be used for these primary analyses.

In addition, the tests will be evaluated over the whole range of cut points by ROC curve analyses and the area under the ROC curve (AUC) will be determined. All analyses will be performed for the total trial population, as well as stratified for gender and separately for the different colonoscopy results (advanced adenomas, defined as presence of at least 1 adenoma with at least 1 of the following features: ⩾1 cm in size, tubulovillous or villous components, high-grade dysplasia, and carcinomas). Analyses will be carried out for stool tests taken at baseline before commencing the study medication and on different days after a single dose of the study drug. Furthermore, analyses will be carried out in which results of tests from stool samples taken on multiple (2 or 3) different days are combined to assess the gain in diagnostic performance by test application on multiple days. Diagnostic performance and its enhancement by acetylsalicylic acid will be evaluated and compared for both of the two iFOBTs employed.

Safety analyses will be performed in the total trial population (i.e. all randomized patients, independent from the availability of stool samples or colonoscopy reports).

The primary endpoints will be analyzed with confirmatory aim; the other statistical analyses will be exploratory.

## Discussion

In this trial we aim to evaluate the diagnostic performance of two iFOBTs for detecting advanced colorectal neoplasms after a single dose of acetylsalicylic acid as compared to placebo. Additionally, gender-specific performance of the tests and gain in diagnostic performance by test application on multiple days will be evaluated.

### Choice of acetylsalicylic acid dose

Most participants in our previous study [7] used acetylsalicylic acid in a dose of 100 mg per day. This dosing scheme leads to predictable suppression of platelet function after repeated administration. However, the onset of effects is slow and maximal suppression is achieved only after about 5 days [15]. Therefore, a loading dose of 500 mg is given in clinical routine when a rapid onset of thrombocyte inhibition is required. Based on published data, a dose of 300 mg seems to be similarly effective [16, 17]. However, an enteric-coated preparation of the same dose was associated with submaximal inhibition [17] and is therefore not used in our trial.

### Benefit/ risk assessment

Acetylsalicylic acid is a very well-known and well-characterized drug that has been on the market for more than 100 years (since 1899). Only one dose is given in this trail and this dose (300 mg) is lower than the normal adult dose required for pain relief (500 mg) and is usually classified as “low-dose”. As such it is approved for the secondary prevention of myocardial infarction (where doses from 75 to 325 mg are used). Furthermore, patients with risk factors (e.g. bleeding diathesis) are excluded. Thus, the occurrence of serious adverse reactions is considered very unlikely.

Previous studies analyzing the association between acetylsalicylic acid use and the performance of FOBTs addressed the potentially hampered specificity due to an increased risk of bleeding from insignificant colonic lesions or from upper gastrointestinal blood loss [18–22]. However, these studies mostly used guaiac-based FOBTs, which are more likely to detect upper gastrointestinal blood, as they respond to the pseudoperoxidase activity of the heme moiety of hemoglobin, which remains quite stable throughout the gastrointestinal tract, whereas the iFOBTs respond to the globin moiety, which is degraded in the gastrointestinal tract [7, 18–21]. A recent observational study on acetylsalicylic acid use and iFOBT performance showed a trend towards an increase in sensitivity (from 51.2 to 66.7%) without loss of specificity in acetylsalicylic acid or NSAID users, but the difference did not reach statistical significance, likely due to a relatively low number of acetylsalicylic acid/NSAID users [20].

Although these results are intriguing, further work is needed for translation of these findings into routine practice of CRC screening. In particular, low-dose acetylsalicylic acid was used chronically for cardiovascular prevention in most cases and by a relatively small subgroup of participants (approximately 10%) in our previous observational study [7]. Although knowledge on pharmacokinetics and pharmacodynamics of acetylsalicylic acid suggests similar effects of a single dose of acetylsalicylic acid (as might be considered for CRC screening) irrespective of cardiovascular risk, this suggestion should be empirically tested in a double-blind placebo-controlled clinical trial, whose design we report here. Furthermore, most users of low-dose acetylsalicylic acid in our previous study were men and data were insufficient to evaluate diagnostic performance among women. Also, our previous study did not allow the assessment of potential further improvement by stool testing on multiple days rather than on a single day.

### Impact

The participants in this trial have no indication for acetylsalicylic acid and no individual benefit from taking one dose of acetylsalicylic acid. However, should this trial show that one dose of acetylsalicylic acid strongly increases the sensitivity of the iFOBTs without relevant loss in specificity, these findings may improve diagnostic procedures in future patients, which might affect also trial patients if repeated screening is required.

To the best of our knowledge, there has been no previous controlled clinical trial investigating the increase in diagnostic accuracy of iFOBTs with acetylsalicylic acid intake in a large target population for CRC screening, i.e. (largely) asymptomatic women and men at average risk of CRC. If the findings from our previous observational study [7] are confirmed in this trial, this intervention would considerably improve the diagnostic accuracy of iFOBTs and thus lead to enhanced early detection of colorectal neoplasms. Thus, the results of this trial may have a large public health impact.

### Additional file

Additional file 1: List of Ethics Committees who were involved in approval of the trial. (DOCX 20 kb)

## Appendix

### Full list of inclusion and exclusion criteria

Inclusion criteria

- Age 40 to 80 years (both males and females; premenopausal women must have a negative pregnancy test before inclusion into the trial, postmenopausal women are defined as women who have not had menstrual bleeding for at least 12 months, or have been surgically sterilized)
- Planned screening or diagnostic colonoscopy
- Able to speak and understand German sufficiently to be able to give written informed consent and comply with the trial requirements

Exclusion criteria

- Factors potentially influencing the primary endpoint
  - Diseases/symptoms
    - Chronic inflammatory bowel disease (e.g. Crohn’s disease, ulcerative colitis)
    - Colonoscopy due to positive fecal occult blood test
    - History of colorectal cancer
    - Angiodysplasia of the colon
    - Anamnestic or observed blood loss per anum
  - Use of any of the following drugs
    - Within 2 weeks before the trial
      - Anticoagulants (including, but not limited to heparin, vitamin K antagonists [e.g. phenprocoumon, warfarin], direct thrombin inhibitors [e.g. dabigatran], or factor Xa inhibitors [e.g. apixaban, rivaroxaban])
      - Antiplatelet drugs (e.g. clopidogrel, prasugrel, ticlopidine)
    - Within 1 week before the trial
      - Acetylsalicylic acid
    - Within 3 days before the trial
      - NSAIDs including selective COX-2 inhibitors
- Factors potentially affecting the safety
  - Any current clinically relevant signs and symptoms, including
    - Signs and symptoms suggesting acute peptic ulcer disease
    - Known clinically relevant thrombocytopenia
    - Acute infection
    - Volume deficit (dehydration)
    - Any currently present allergy with dermal reactions, pruritus, or urticaria
    - Severe or insufficiently controlled asthma
    - Severe kidney or liver diseases (e.g. GFR < 30 ml/min, liver cirrhosis)
    - Severe, not sufficiently treated heart failure (as judged by the investigator)
    - Severe, poorly controlled arterial hypertension
    - Any other unclear symptoms needing further investigation in the opinion of the investigator
  - Any of the following anamnestic findings
    - History of severe gastrointestinal bleeding
    - Known hemorrhagic diathesis, including, but not limited to, hypoprothrombinemia, severe thrombocytopenia, hemophilia
    - Asthma, except for patients who have used acetylsalicylic acid in the past without negative effects
    - Hypersensitivity against salicylic acid or other ingredients of the study drugs
    - Previous intolerance to NSAIDs including selective COX-2 inhibitors, or antirheumatic medications
    - Severe gout (e.g. recurrent attacks)
    - Hereditary oxaluria
    - Known G6PD or glutathione peroxidase deficiency
    - Known epilepsy with generalized seizures
    - Severe cardiac diseases (including, but not limited to, myocardial infarction in the past 6 months)
  - Intention to use any of the following drugs during the trial
    - Anticoagulants
    - Antiplatelet drugs
    - NSAIDs including selective COX-2 inhibitors
    - Methotrexate ≥15 mg/week
    - Systemically administered glucocorticoids
    - Selective serotonin reuptake inhibitors (SSRIs)
    - Valproic acid
  - Planned surgery/dental treatment during participation in the trial
- Other factors
  - Known or suspected relevant alcohol abuse
  - Known or suspected illicit drug abuse
  - Pregnancy
  - Suspected non-compliance with the trial procedures
  - Participation in another clinical trial

## Abbreviations

AE: Adverse event
AMG: German Drug Law (Deutsches Arzneimittelgesetz)
AUC: Area under the curve
BfArM: Bundesinstitut für Arzneimittel und Medizinprodukte
CRC: Colorectal cancer
GCP: Good clinical practice
GCP-V: Good clinical practice regulation (GCP-Verordnung)
GMP: Good manufacturing practice
ICH: International conference on harmonisation of technical requirements for registration of pharmaceuticals for human use
iFOBT: Immunochemical fecal occult blood test
ISF: Investigator site file
LKP: Principal/Coordinating Investigator according to AMG (Leiter der Klinischen Prüfung)
ROC: Receiver operating characteristics
SAE: Serious adverse event
SC: Steering committee
