# Supplementary material for: Clinical trial protocol of the ASTER trial: a double-blind, randomized, placebo-controlled phase III trial evaluating the use of acetylsalicylic acid (ASA) for enhanced early detection of colorectal neoplasms
Source: BMC Cancer. 2018 Sep 24;18:914. doi: 10.1186/s12885-018-4826-3 (PMC6154882; doi:10.1186/s12885-018-4826-3)
Supplement: Supplementary file 1 — List of Ethics Committees who were involved in approval of the trial. (DOCX 20 kb) [file 12885_2018_4826_MOESM1_ESM.docx]

**Additional File 1.**

Supplement 1: List of Ethics Committees who were involved in approval of the trial

Responsible Ethic Committee (“Federführende Ethikkommission”):

- Ethics Committee of the Medical Faculty of the University of Heidelberg (Ethikkommission der Medizinischen Fakultät Heidelberg), Alte Glockengießerei 11/1, 69115 Heidelberg

Names of the Ethics Committees responsible for the recruitment centers (“Beteiligte Ethikkommissionen”):

- Medical Board in North Rhine (Ethikkommission der Ärztekammer Nordrhein), Tersteegenstraße 9, 40474 Düsseldorf
- Medical Board of the State Chamber of Medicine in Rheinland-Palatinate (Ethik-Kommission der Landesärztekammer Rheinland-Pfalz), Deutschhausplatz 3, 55116 Mainz
- Medical Board of State Medical Council of Baden-Württemberg (Ethik-Kommission bei der Landesärztekammer Baden-Württemberg), Jahnstraße 40, 70597 Stuttgart
- Medical Board of State Medical Chamber of Saxony (Ethikkommission bei der Sächsischen Landesärztekammer), Schützenhöhe 16, 01099 Dresden
- Medical Board of the Bavarian State Medical Chamber (Ethik-Kommission der Bayerischen Landesärztekammer), Mühlbaurstraße 16, 81677 München
- State Medical Association in Saarland (Ethik-Kommission bei der Ärztekammer des Saarlandes), Hafenstraße 25 66111 Saarbrücken
- Medical Board of the Department of Medicine at the Johann Wolfgang Goethe University Frankfurt am Main (Ethik-Kommission des Fachbereichs Medizin der Johann Wolfgang Goethe-Universität Frankfurt am Main), Theodor-Stern-Kai 7, Haus 1 60590 Frankfurt

Competent authority for the approval of clinical drug trials in Germany:

- The Federal Institute for Drugs and Medical Devices (Bundesinstitut für Arzneimittel und Medizinprodukte, BfArM), Kurt-Georg-Kiesinger-Allee 3, 53175 Bonn
